# Ultrasound-Assisted Extraction of Protein from *Moringa oleifera* Seeds and Its Impact on Techno-Functional Properties

**DOI:** 10.3390/molecules28062554

**Published:** 2023-03-11

**Authors:** Khushar Fatima, Muhammad Imran, Muhammad Haseeb Ahmad, Muhammad Kamran Khan, Waseem Khalid, Ammar AL-Farga, Wafa S. Alansari, Ghalia Shamlan, Areej A. Eskandrani

**Affiliations:** 1 Department of Food Science, Faculty of Life Sciences, Government College University, Faisalabad 38000, Pakistan; 2 University Institute of Food Science and Technology, The University of Lahore, Lahore 54000, Pakistan; 3 Biochemistry Department, Faculty of Science, University of Jeddah, Jeddah 21577, Saudi Arabia; 4 Department of Food Science and Nutrition, College of Food and agriculture Sciences, King Saud University, Riyadh 11362, Saudi Arabia; 5 Chemistry Department, Faculty of Science, Taibah University, Medina 30002, Saudi Arabia

**Keywords:** *Moringa oleifera*, seed, protein, ultrasound, functional properties

## Abstract

Plant proteins can be an important alternative to animal proteins subject to minor modification to address sustainability issues. The impact of ultrasound application on the yield, techno-functional properties, and molecular characteristics of protein extracted from *Moringa oleifera* seeds was studied. For this purpose, a central composite design (CCD) was applied to optimize ultrasound-assisted extraction (UAE) parameters such as amplitude (25–75%), solute-to-solvent ratio (1:10–1:30), and pH (9–13) for obtaining the maximum protein yield. At the optimized conditions of 75% amplitude, 1:20 solute-to-solvent ratio, and 11 pH, a protein yield of 39.12% was obtained in the UAE process. Moreover, the best sonication time at optimized conditions was 20 min, which resulted in about 150% more extraction yield in comparison to conventional extraction (CE). The techno-functional properties, for instance, solubility, water (WHC)- and oil-holding capacity (OHC), and emulsifying and foaming properties of the protein obtained from UAE and CE were also compared. The functional properties revealed high solubility, good WHC and OHC, and improved emulsifying properties for protein obtained from UAE. Although protein from UAE provided higher foam formation, foaming stability was significantly lower.

## 1. Introduction

Proteins impart some important techno-functional properties such as emulsification, foaming, and/or gelling, owing to which they are considered to be one of the main components of food products [1]. Proteins with specific functionalities are either synthesized chemically or extracted from animal and plant sources. Due to the intensive competition among industries and more specified demands from the consumers and to address sustainability issues, food industries always look for solutions to meet market challenges. Currently, plant-derived proteins, particularly from agro-industrial waste, receive substantial attention as a sustainable alternative to animal-based proteins due to the rising cost of animal proteins and food security and sustainability issues [2].

*Moringa oleifera* (Lam.), which is a widely cultivated species in native parts of Asia and Africa, belongs to the family *Moringaceae* [3]. *M. oleifera*, also known as Drumstick tree, is considered to be a nutritionally dense plant and is also referred to as a ‘miracle Tree’ because of its multi-purpose uses. Ease of cultivation makes it a cheap source of high-quality nutrients and ingredients in traditional herbal medicines [4]. The extract of its leaves is rich in important phytochemicals and has potential as an antioxidant [5], anti-microbial [6], anti-inflammatory [7], and anticancer agent [8]. Although seeds of *M. oleifera* are rich in protein (35–36%) and oil (38–39%) [9], they are still used mainly as feedstock [10] and currently have no value-added ingredients or products. The present study revolves around the extraction of *M. oleifera* seed protein (MOSP) to develop a high value-added ingredient for food products.

The main challenge while extracting the proteins is to choose the appropriate extraction technique. Traditionally, methods such as alkaline, organic solvent, salt, and enzymatic extraction, which are used for protein extraction, are less efficient, require long extraction time, give less protein yield, involve a high amount of solvents, and ultimately lead to an environmental burden [11]. The application of novel and more accurate protein extraction techniques can enhance the functional properties of foods. Ultrasonic-assisted extraction is considered to be an efficient and environmentally friendly extraction technique compared to conventional and other novel techniques because of its lower extraction time, high extraction yield, low solvent consumption, and enhanced functional properties of protein [12]. Sonication technique is associated with the phenomenon of acoustic cavitation, during which the collapse of bubbles releases energy for enhanced mass transfer from and to the interface; thus, this technique is classified as a sustainable and green technique [13]. The extraction efficiency of ultrasound is influenced by different parameters, such as ultrasonic intensity, solute-to-solvent ratio, treatment time, temperature, etc., which need to be optimized using appropriate combinations or statistical designs [14].

Conclusively, the study is mainly focused on the effect of techno-functional properties such as protein solubility, water- and oil-holding capacity, and emulsifying and foaming properties of *M. oleifera* seed protein extracted via the sonication technique. Moreover, fluorescence and Fourier-transform infrared spectra will be taken to identify changes in functional groups.

## 2. Results and Discussion

### 2.1. Optimization of Ultrasonic-Assisted Extraction (UAE) of Protein

#### 2.1.1. Fitting the Proposed Model

Response surface methodology (RSM) is a useful statistical technique for evaluating the influence of different factors and for designing experiments such as the one in this study [15]. An empirical model can be designed to evaluate the optimized conditions using the required response. In the present study, the effect of ultrasonication on the extraction efficiency of *M. oleifera* seed protein (MOSP) was evaluated to observe the maximum recovery of the protein using RSM. For this, a central composite design (CCD) was applied using three independent variables: amplitude (%) (A), solute-to-solvent ratio (g/mL) (B), and pH (C) (Table 1) with 17 runs with 3 central points. Usually, if one ultimately misses any runs, the accuracy of the remaining runs in the Box–Behnken Design (BBD) becomes critical to the dependability of the model, so a CCD is preferred.

The MOSP yield obtained ranged between 30 and 39%, depending on the combination of the trial. Experimental run 16 showed the lowest yield with an amplitude of 25%, a solute-to-solvent ratio of 1:10, and a pH of 9, while experimental run 10 showed the highest yield with an amplitude of 75%, a solute-to-solvent ratio of 1:20, and a pH of 11. The difference between the measured values and the predicted values is minimal, which shows the rationality of the measured values. Following quadratic polynomial regression, an equation of amplitude (A), solute-to-solvent ratio (B), and pH (C) was developed to obtain the response model of extraction yield:

*Y*_1_ = 36.59 + 3.08 A + 0.4920 B + 0.1120 C + 0.0050 AB + 0.0050 AC − 0.0250 BC − 0.3726 A^2^ + 0.1124 B^2^ − 1.71 C^2^

The results obtained were further processed for analysis of variance (ANOVA) with a 95% confidence level in order to check the significance and suitability of the response model (Table 2).

The results indicate that the *p*-value of the overall model is less than 0.0001, which is highly significant, and it indicates the sustainability of the fitted model. Additionally, the F-value documented the significance of the response model. Moreover, the value for determination coefficient (*R*^2^) was 0.9999, and the adjusted value (*R*^2^ adjusted) for the extracted yield was 0.9998, which shows that the model was correctly interpreted using the measured data. Generally, *R*^2^ values higher than 0.75 are considered to be superlative for a good fitted model [16]. Furthermore, the values of the coefficient estimate report the expected change in the response factor value when all remaining factors are held at a medium level (Table 3). Coefficients with positive values represent a linear increase in the response factor, and those with negative values document the linear decrease in the dependent factor. The accuracy of the model was checked via the correlation coefficient.

#### 2.1.2. Single-Factor Analysis for Protein Yield

**Figure 1:**
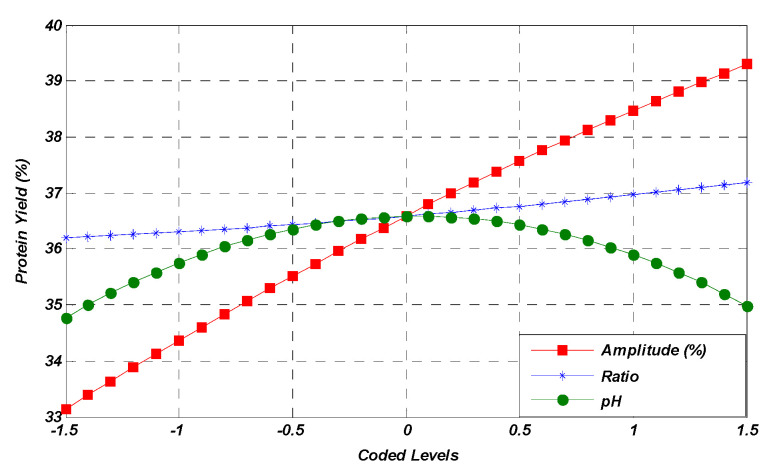
Linear effect of studied parameters on the MOSP yield.

According to the results obtained, it was observed that the amplitude (A), solute-to-solvent ratio (B), and pH (C) independent factors had a significant effect on the yield of MOSP. The variation in the response factor (yield, %) due to independent variables in the form of coded levels is also presented in Figure 1. The effect of the individual variable was noted while keeping the other two at their medium values. The single-factor response graph shows that the amplitude variable has a direct effect on the increase in yield. On the other hand, the pH imparts an increasing trend for the response yield until the central point, while a decrease in pH leads to a low yield of response factor after the central point. Moreover, the independent solute-to-solvent ratio factor has shown a non-significant impact on the response factor before and after the central point.

#### 2.1.3. Effect of Mutual Interactions on Protein Yield

**Figure 2:**
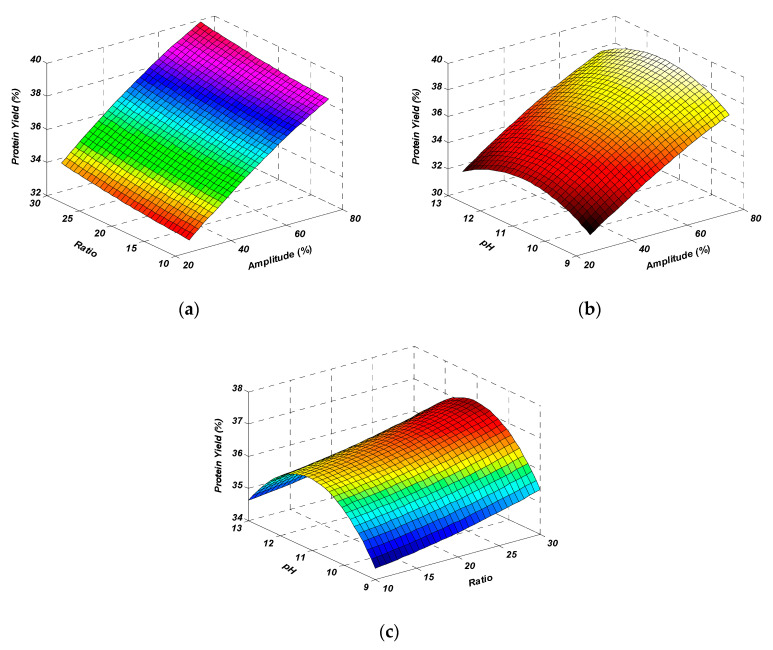
Response surface plots representing the effect of mutual interactions of studied parameters on the protein yield. Interaction between amplitude & ratio (**a**), amplitude & pH (**b**) and ratio & pH (**c**) while keeping third parameter at central value.

The interactions that occurred between amplitude and solute-to-solvent ratio (AB), amplitude and pH (AC), and solute-to-solvent ratio and pH (BC) had a non-significant effect on protein yield (*p* > 0.05). The interaction between independent variables is also shown in the form of 3D response surface plots (Figure 2). Through the study of the obtained results, the maximum yield obtained by the mutual interaction of amplitude and solute-to-solvent ratio (Figure 2a) was observed. It was observed from the results that when interaction between amplitude and pH occurred with the average value of the solute-to-solvent ratio, the yield percentage slightly decreased at the maximum amplitude (Figure 2b). On the other hand, there was a decrease in the yield percentage as the solute-to-solvent ratio and pH were considered to be interacting variables by taking the average fixed amplitude (Figure 2c). These plots showed that the selected factor levels were logical enough and had a positive influence on extraction yield. According to the figures, the interactions between the variables significantly affected the extraction yield, which was also interpreted through ANOVA (Table 2).

#### 2.1.4. Optimization and Validation

**Figure 3:**
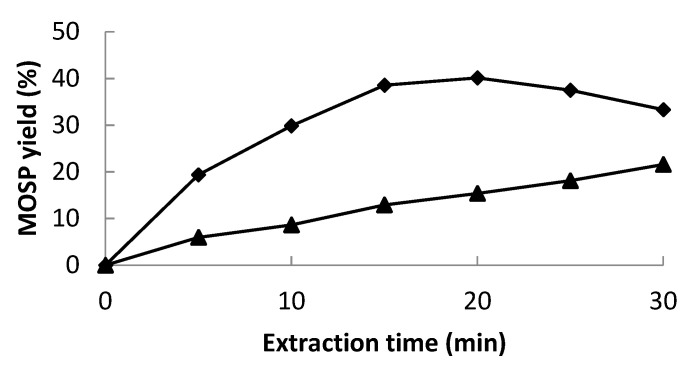
MOSP yield (%) during ultrasound-assisted extraction (◆) and conventional extraction (▲).

The predicted extraction yield at optimized conditions was noted from the experimental design. According to CCD and contour plots, the best extraction yield (39.90%) was predicted at the optimized conditions of an amplitude higher than 75%, a solute-to-solvent ratio of 1:22, and a pH of 11.37, which were rounded off to 75%, 1:20, and 11, respectively, for the ease of experimentation. Validation of the statistical model and the regression equation was confirmed by repeating the experimental run at defined optimized conditions. The measured value of the extraction yield (39.12%) at optimized conditions (amplitude 75%, solute-to-solvent ratio of 1:20, and pH 11) confirmed the validity of the model. The obtained values were quite close to the predicted ones. Overall, the results showed that the model for the extraction of MOSP through ultrasonication was fit and acceptable. Furthermore, a comparison of protein yield was made between UAE and conventional extraction (CE). While keeping the other parameters at optimized levels, the sonication treatment resulted in a 150% increase in the protein yield in comparison to CE (Figure 3).

For a commercial point of view, the yield of protein is highly important, particularly for the efficient utilization of agro-waste and to achieve the goal of sustainable development. Conventional extraction techniques for protein other than ultrasonication are either time-consuming or less productive. Therefore, UAE was applied for the extraction of protein from *M. oleifera* seeds. As it was noticed during this study, various studies have also shown an increase in the protein yield with the increase in the power/amplitude of the sound waves, for instance, wampee seed protein [11], lupin seed protein [17], *Eurycoma longifolia* root protein [18], and *Dolichos lablab* L. bean protein [19]. It is known that ultrasonication works on the principal of cavitation, and as a result of the cavitational effect, a mechanical force is generated which helps in the transport of protein across the cell barrier, either by increasing the flow of solvent on both sides or, sometimes, by rupturing the cell barrier [20]. Although ultrasonic amplitude remained promising for a high MOSP yield, the solute-to-solvent ratio presented a minor effect on the protein yield. The lower solute-to-solvent ratio led to the lower difference in protein concentration inside and outside the cell matrix, thus reducing the protein yield [11]. On the other hand, the higher solute-to-solvent ratio resulted in less ultrasonic energy density per unit volume and ultimately, lower protein yield was observed. Although there was a minimal effect of the solute-to-solvent ratio on the MOSP yield, the optimum value of 1:20 g/mL was found to be best for the maximum MOSP yield. The solute-to-solvent ratio often varies depending on the protein source, the structure of the protein, the extraction medium, and others [21]. The pH value significantly affected the protein yield, as the protein yield increased with the increase in pH value up to 11. This might be attributed to the breakage of H-bonds in the cell matrix and, thus, an increase in the protein yield [22]. Any further increase in pH resulted in the degradation of protein, causing reduced solubility, and consequently, the yield of MOSP decreased. Similar results were obtained during the extraction of proteins from wampee seed [11] and *Dolichos lablab* L. [19].

At optimized conditions, different ultrasonic times (0–30 min) were observed for the maximum MOSP yield, and a higher yield was found at 20 min (Figure 3). With further increases in time, a slight decrease was observed. The 30 min treatment was counterproductive by dropping the protein yield. This might be due to the structural degradation of protein, which generally develops aggregates by folding to resist the extreme conditions, and ultimately, proteins do not solubilize and possibly finish with the centrifugation residue [23]. In the case of conventional extraction, the yield of MOSP remained less than that of UAE at each time period, which further confirms the effectiveness of the sonication treatment. In most of the studies based on the sonication treatment for the extraction of protein from different plant sources, the best extraction time ranged between 15 and 20 min [21,23,24]. The time may increase depending on the ultrasound intensity and equipment; for instance, this ultrasound-assisted extraction time may reach more than 60 min if an ultrasonic cleaner was used rather than an ultrasonic probe [25].

### 2.2. Functional Properties of MOSP

#### 2.2.1. Solubility

Solubility is one of the most important functional properties of protein. It is the thermodynamic property which can be elaborated as the ‘protein concentration in a saturated solution’, and it must be at equilibrium with the solid phase at optimal conditions. Solubility of proteins can be altered by any extrinsic and intrinsic factors [26]. At neutral pH, the solubility of MOSP extracted via the conventional method was 5.56 ± 0.13%. A significant increase was observed in the solubility of MOSP extracted with the ultrasonic method, which was 29.82 ± 0.21% (Table 4). Ultrasonication of MOSP resulted in the enhancement of protein solubility. This may have happened because of the reduction in particle size of the MOSP alteration in the molecular structure and changes in the conformation [27], thus exposing more hydrophilic groups in the medium for increased solubility. Similar results were observed on the solubility of whey protein [28] and pea protein [29].

The high amplitude level of ultrasound escalates the protein solubility by altering the conformation and structure of protein; this results in the inside aperture of hydrophilic ends of amino acids toward water [30]. A larger area of protein was covered up with water, as the molecular weight of the treated protein was reduced due to the high ultrasonic amplitude [31]. The temperature rise due to ultrasonication also had a significant effect on the improvement of protein solubility, as protein solubility rose with the rise of temperature, as reported in the studies for soy protein [32]. Enhancement in protein solubility could also be due to the alteration in the three-dimensional structure of globular protein, which resulted in a higher number of charged groups established with high electrical conductivity, unlike the CE sample. Under those conditions, as more water interacted with proteins, and electrostatic forces increased, the inter-linkage between water and protein improved, thus increasing the protein solubility.

#### 2.2.2. Water (WHC)- and Oil-Holding Capacity (OHC)

WHC and OHC are important functional properties of protein used for the moderation of the texture and viscosity of the food product and also for the reduction in the processes of dehydration during food storage [33]. The ability of proteins to retain or hold water in their three-dimensional structure is known as the WHC of protein. Proteins with a high WHC during application in a food product can dehydrate the other ingredients present in food, and thus, the product becomes less sensitive to storage humidity [34].

The WHC of MOSP extracted with CE was 0.86 ± 0.009 g/g, and that of the UAE sample was 1.02 ± 0.006 g/g (Table 4). The increase in water-holding capacity after ultrasonication was probably due to the spongy structure generated by peptide chains and due to the ionized polarity groups formed after ultrasound treatment [35]. Because of these groups, loose structures were formed and produced more space for water storage; thus, this condition resulted in a higher WHC. Similar results were obtained after the application of ultrasonication on the beef *Longissmus lumborum* [36].

Oil-holding capacity (OHC) is the ability of proteins to trap oil or fat within their non-polar chains. The oil-holding capacity of the MOSP sample extracted with CE was 0.91 ± 0.015 g/g (Table 4). According to the results obtained, the OHC of the MOSP extracted with UAE was higher than that of the CE one. The ultrasonically treated MOSP sample had a significantly higher OHC (1.91 ± 0.013 g/g). Ultrasonication increased the surface exposure of hydrophobic groups, due to which a strong linkage formed with triglyceride molecules, resulting in the improved OHC (Boukhari, Doumandji et al. 2018). The same trends were observed in related studies on whey protein isolates [16] and tamarind seeds protein isolates [37].

The improved WHC and OHC are considered helpful for enhancing the shelf life of processed foods given the fats and water reduced from the surface [38]. Their higher values also improve the mouth feel of the product. Overall, it was observed that ultrasonic treatment had a significant impact on water- and oil-holding capacities.

#### 2.2.3. Emulsion Capacity and Emulsion Stability

The emulsifying property of protein is its ability to form an emulsion and to maintain the stability of the newly formed emulsion [39]. It is an important parameter in the production of various fabricated foods. The emulsion capacity of MOSP extracted with CE was 58.39 ± 1.68 mg/mL (Table 4). Next, the ultrasonic treatment emulsion capacity of MOSP significantly increased to 75.93 ± 1.19 mg/mL. Prepared emulsions were kept at room temperature to calculate the emulsion stability. A slight improvement was observed in the emulsion stability of the MOSP extracted with UAE (W = 4%) as compared to that of the CE one (W = 6%); this improvement might be associated with the higher hydrophobic levels and increased droplet size of oil in the water emulsion, which was established via ultrasonication [40]. In addition, it has been reported that the increased solubility of proteins resulted in a maximal emulsifying capacity. Furthermore, the increased emulsifying capacity of MOSP might be related to the alternation in aggregation, solubility, and secondary structure [41]. A recent study which was conducted to study the effects of ultrasonication on animal and vegetable proteins showed similar development in the emulsion properties of protein [42,43].

#### 2.2.4. Foaming Capacity and Foaming Stability

The total inter-facial area formed by the whipping of protein is called the foaming capacity (FC) of the protein [44]. The stability of foam is calculated as the total time required to lose the volume of the foam. It is an important functional property of protein that helps in the production of formulated foods. The foaming capacity of MOSP extracted with CE was 13.21 ± 0.27% (Table 4). The foaming capacity of MOSP was improved to 24.23 ± 0.64% after ultrasonic treatment. The observed enhancement in foaming capacity might be associated with the effect of ultrasonic homogenization, which improved the foaming power [45]. This homogenization evenly distributed the particles of protein, which increased the foaming ability of the MOSP sample extracted with UAE. Corresponding results were obtained in another study on whey protein [46]. The increased exposure of hydrophobic groups aided in the dispersion of protein molecules toward air-water interface and their surface assimilation on it. A similar trend in the foaming capacity of protein was also shown in an earlier study conducted to study the effects of high-intensity ultrasonic treatment on food proteins [47]. However, the trend was opposite in the case of foaming stability (FS). There was no significant improvement observed in MOSP extracted with UAE when compared to the CE one. The reason for this might be the ultrasonic cavitation which increased MOSP solubility, and the foaming stability dropped because of the decrease in surface activity [27]. Furthermore, the breakdown of larger peptide units into smaller ones also causes the reduction in foaming stability. It was reported earlier in a few studies that ultrasonication may have had no significant effect on foaming stability, as observed in de-hulled yellow mustard protein [48] and pea protein [49] isolates.

### 2.3. Structural Study of MOSP

#### 2.3.1. FT-IR Analysis

**Figure 4:**
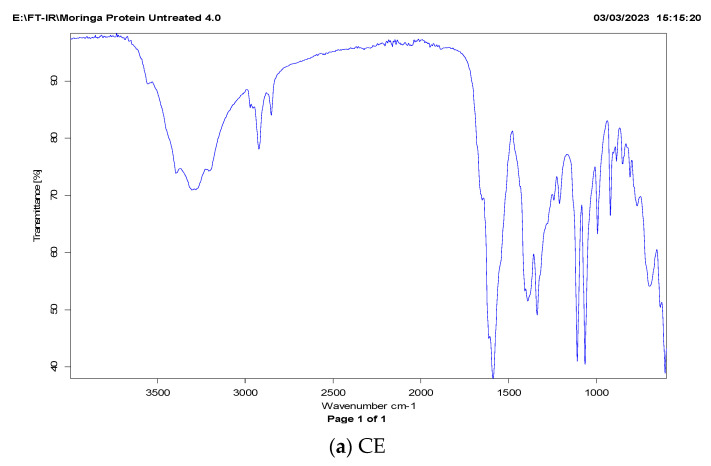

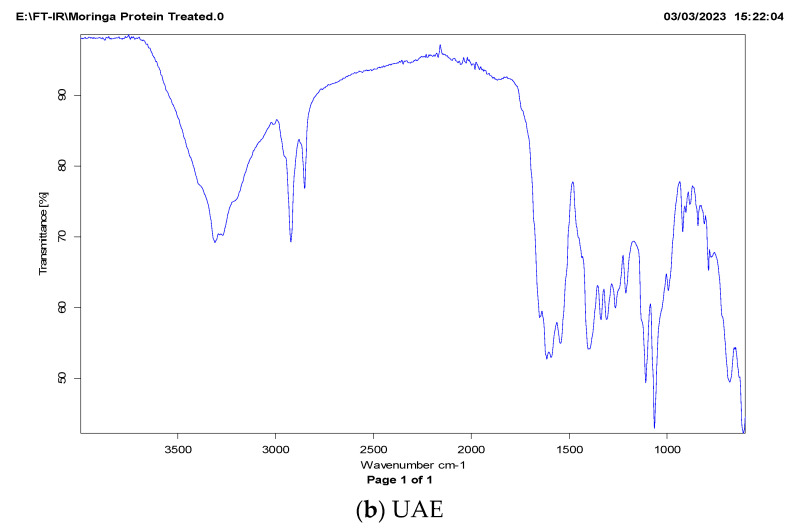
FT-IR spectra of *M. oleifera* seed protein (MOSP) isolate from conventional extraction (**a**) and ultrasonic-assisted extraction (**b**).

The FT-IR spectrum of MOSP can be described in three importance wavelength bands (Figure 4). Generally, the FT-IR spectrum is based on the different amide zones [50]. The amide-I zone representing C=O bonds is stretched over the wavelength range from 1700 to 1600 cm^−1^; the amide-II zone is associated with N–H bonds which are spread over the 1575 to 1480 cm^−1^ wavelength range; and the amide-III zone representing the N–H bending and C–N is stretched over range of 1400–1200 cm^−1^. Among these bands, the amide-I is the most responded area to any chemical change in the secondary structure of protein. The FT-IR spectra of MOSP isolated via conventional extraction and ultrasonic-assisted extraction are shown in Figure 4. It can be observed that sonication significantly affects the amide-I region based on C=O stretching, while only a change in the intensity of peaks was observed for other regions. A similar trend was observed on quinoa seed protein isolates [51] and *Moringa oleifera* seed protein isolates [52] treated with sonication.

#### 2.3.2. Intrinsic Fluorescence Patterns

**Figure 5:**
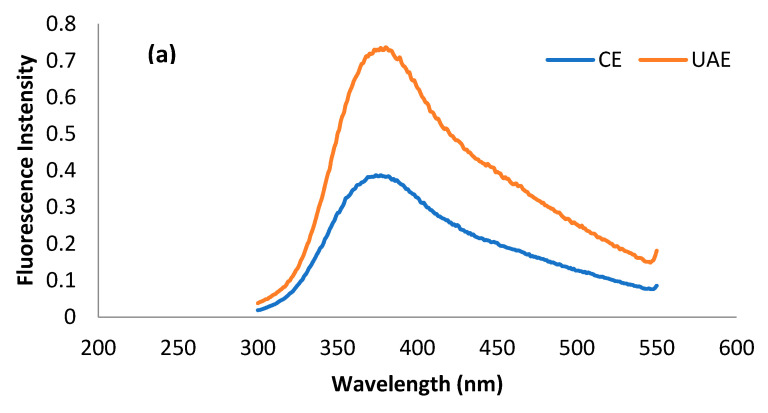

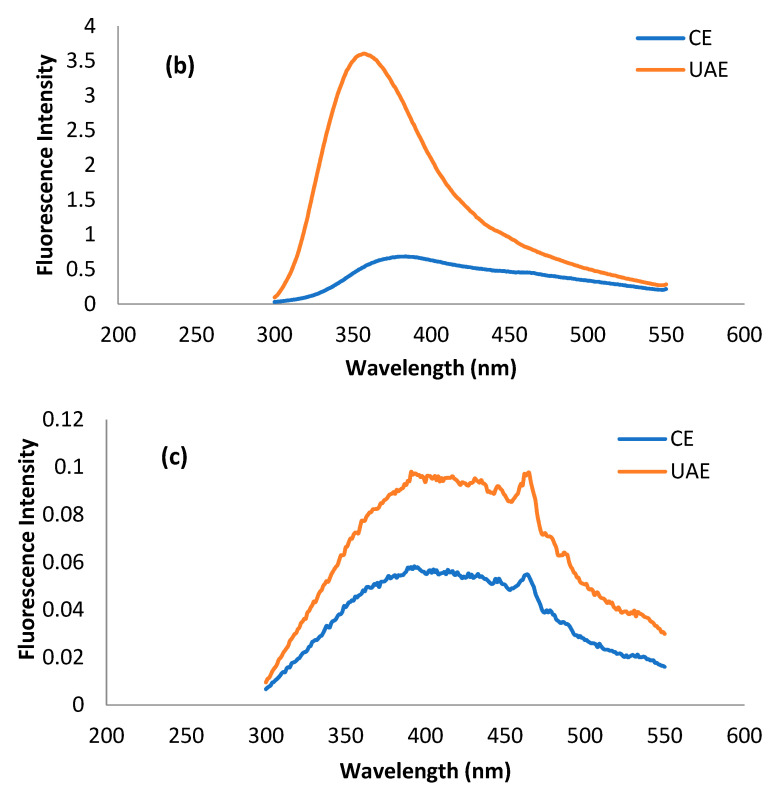
Fluorescence spectra of tryptophan (**a**), phenylalanine (**b**), and tyrosine (**c**) residues for the MOSP from CE and UAE.

The fluorescence spectrum is basically a representation of amino acid residues such as tryptophan, tyrosine, and phenylalanine present in a protein, which are, in principal, detrimental to the tertiary structure of protein. Most of the studies related to tertiary structure using intrinsic fluorescence are based on the changes in tryptophan intensity [52]. Usually, λ_max_ of tryptophan < 330 nm shows the occurrence of the amino acid in the non-polar environment, while λ_max_ > 330 nm shows its presence in the polar environment due to conformational changes in the tertiary structure of protein [51]. The presence of tryptophan, tyrosine, and phenylalanine residues in MOSP is evident from previous studies [53,54]. The fluorescence spectra of tryptophan, tyrosine, and phenylalanine residues for the protein obtained from conventional extraction and ultrasound-assisted extraction are shown in Figure 5. It can be observed that λ_max_ for tryptophan, phenylalanine, and tyrosine in the case of MOSP from CE was observed at 380 nm, 380 nm, and 390 nm. After MOSP was obtained by using UAE, no shift in λ_max_ was found except for phenylalanine, which shifted to 360 nm. Moreover, an increase in the fluorescence intensity was found in all of the studied amino residues. The spectra of tyrosine give some noise, but they could be helpful for observing the considerable changes in the chemical structure of MOSP.

The increase in the intensity of amino residues is related to the oxidation of protein by hydroxyl radicals produced during the sonication treatment [20]. A similar trend was observed in the case of lupin protein [21] and pea protein [17]; however, the observation was in conflict with the results of quinoa seed protein isolates [51] and walnut protein isolates [55], which experienced a decrease in the intensity from the application of ultrasound. These discrepancies are related mainly to the production of different amounts of hydroxyl radicals due to the variation in the intensity of ultrasound treatment, the duration of the treatment, and the concentration and types of substrates of treated proteins. Unfortunately, no study was found to provide correlating results for phenylalanine and tyrosine. Nevertheless, this increase in the intensity could be attributed to the response of different amino acid residues to the variety of stresses to the protein due to the structure of the amino acid residues [56].

## 3. Material and Methods

### 3.1. Raw Materials and Chemicals

**Figure 6:**
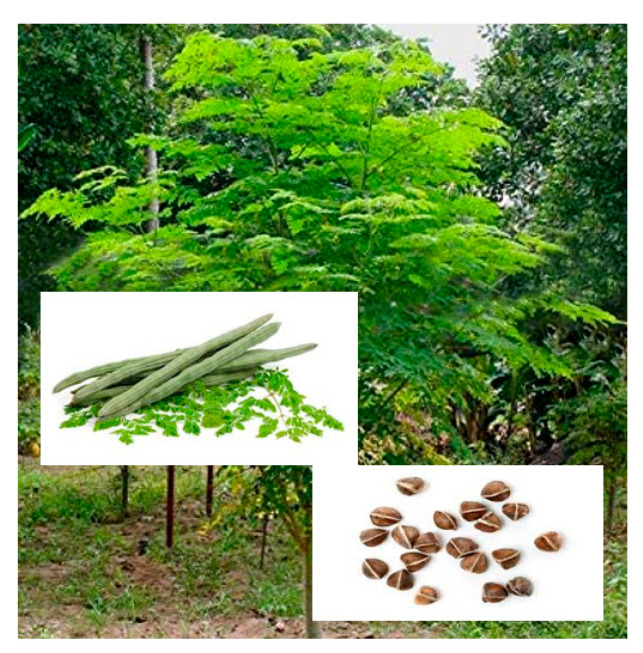
Moringa plant, pods, and seeds.

*M. oleifera* seeds (Figure 6) were procured from Ayub Agriculture Research Institute, Faisalabad, Pakistan. Dried *M. oleifera* seeds were milled in a commercial grinder and then passed through a 100-mesh sieve to obtain fine seed powder. Peanut and corn oils were purchased from a local market. Coomassie brilliant blue G-250 and bovine serum albumin (BSA) of analytical grade were acquired from Sigma-Aldrich. The rest of the chemicals, such as sodium hydroxide, hydrogen chloride, acetone, methanol, and others, were obtained from Duksan, Korea.

### 3.2. Ultrasonic-Assisted Extraction (UAE) of Seed Protein

The UAE method of [18] was adopted with slight modification. Briefly, for each extraction, samples with varying solute-to-solvent ratios (1:10, 1:20, and 1:30) at different pH levels (9, 11, and 13) were sonicated using a 13 mm probe [57] for 15 min at a frequency of 20 kHz and a net output power of 750 W but with variable amplitudes (25, 50, and 75%) using sonication apparatus VCX750 (Sonics & Materials, Inc. Newtown, CT, USA). In total, 17 runs were performed, as per response surface statistical design (see Table 1). After optimization of amplitude, solute-to-solvent ratio, and pH, the best extraction time was determined by performing trials for 0, 5, 10, 15, 20, 25, and 30 min at optimized conditions. The conventional extraction (CE) of protein at optimized conditions but without amplitude was also performed for the comparison and validation of the sonication application.

### 3.3. Protein Quantification by Bradford Method

The amount of protein was determined by using the Bradford method [58]. Concisely, after sonication/conventional extraction, each sample was centrifuged (Thermo Scientific Heraeus Megafuge 8R-Germany) at 4 °C at 7508× *g* for 20 min, and the supernatant was collected and diluted. Then, 1 mL from each diluted solution was mixed with 5 mL of Coomassie brilliant blue G-250 solution, and the mixture was kept for 2 min at room temperature. The absorbance of each mixture was taken at 525 nm by a UV-Vis Double-Beam spectrophotometer (Specord-200 Plus, Analytik Jena, Germany). Bovine serum albumin (0–100 ppm) was used to prepare a standard curve for quantifying the protein contents of the extracts. Based on the protein content of the extract, the protein extraction yield (g/100 g sample) was calculated using following formula:

Extraction yield (%) = (Protein weight/powder weight) × 100(1)

### 3.4. Isolation of Seed Protein

Protein from the supernatant obtained during UAE/CE under optimized conditions was isolated by following acid–base methodology [59]. Briefly, the pH of the supernatant was adjusted to 3, which is the isoelectric point for seed protein, and the solution was stored at 4 °C for 6 h. Precipitates were then collected on filter paper from each sample by washing them twice with distilled water. These precipitates were then dispersed in deionized water, followed by pH adjustment to 7. Finally, solutions were freeze-dried for 48 h to obtain *M. oleifera* seed protein (MOSP).

### 3.5. Functional Properties of M. oleifera Seed Protein (MOSP)

#### 3.5.1. Solubility

The solubility of the protein samples was estimated by using the method described by [33]. For this, 10 mg of MOSP was dispersed in 8 mL of deionized water, and the pH was adjusted to 2–10 (if necessary) with either 1.0 M NaOH or HCl. The protein solution was then stirred at room temperature for 30 min. The volume of the solution was then adjusted to 10 mL by adding the respective pH solutions. These solutions were then centrifuged for 20 min at 7508× *g*. After the determination of the protein content in the supernatant via the Bradford method, protein solubility was calculated via the following formula:

Solubility (%) = (Protein content in supernatant/Total protein in sample) × 100(2)

#### 3.5.2. Water- and Oil-holding Capacity

The method presented by Yılmaz and Hüriyet [60] and Saha and Deka [61] was adopted to determine the water (WHC)- and oil-holding capacity (OHC) of samples. The determination of the WHC/OHC of the samples was performed by mixing 0.3 g from each sample with 5 mL of deionized water or peanut oil in centrifuge tubes. The mixtures were vigorously vortexed and left for 30 min at room temperature. The mixture solutions were centrifuged at 3003× *g* for 15 min. The calculation of WHC and OHC for each sample was performed according to the following formula:

WHC or OHC (g/g) = (W2 − W1)/W0(3)

where W0 = weight of dry sample in grams, W1 = weight of dry sample and tube, W2 = weight of sediments and tube.

#### 3.5.3. Emulsion Capacity and Emulsion Stability

The emulsion capacity (EC) and emulsion stability (ES) of samples were determined according to the method of Jiang, et al. [62]. The EC of the samples was determined by dissolving 1 g from each sample with 50 mL of 0.1 NaOH in a 250 mL beaker. Then, 50 mL of corn oil was added, and the mixture was homogenized (FSH-2A Homogenizer, Changzhou, China) at 10,000 rpm for 2 min to form an emulsion. The emulsion was then transferred into a 100 mL measuring cylinder. The EC was determined by calculating the difference between the initial volume (Vi) of oil and the released volume (Vr) of oil against the weight of the sample (*W*), which were taken as shown in the following formula:

EC = (Vi − Vr)/*W* (4)

The emulsion stability (ES) was observed after 48 h at room temperature by determining the amount of separated water from oil.

*W* (%) = Vol. of separated water (mL)/Original amount of water (mL) × 100 (5)

*W* is the percentage of water separated.

#### 3.5.4. Foaming Capacity and Stability

The method adopted by Phongthai, et al. [63] for the determination of foaming capacity (FC) and foaming stability (FS) was used for the purpose. To measure FC and FS, 50 mL of 0.1% (*w*/*v*) from each protein solution was taken in a 150 mL beaker and homogenized (FSH-2A Homogenizer, China) for 1 min at 24,000 rpm. The total volume was measured at 0 and 10 min. FC and FS were calculated according to the following formulae:

FC (%) = (V1 − V0)/V0 × 100(6)

FS (%) = (V2 − V0)/V1 − V0 × 100(7)

where V0 is the volume of the protein solution before homogenization; V1 is the volume of the protein solution after homogenization at 0 min; V2 is the volume of the protein solution after homogenization at 10 min.

### 3.6. Fourier-Transform Infrared (FT-IR) Spectroscopy

FT-IR is considered to be one of the best techniques for the determination of the secondary structure of proteins [51]. In this study, spectra to assess the composition of a protein’s secondary structure was acquired by an infrared spectrometer (Alpha II FT-IR, Bruker, Billerica, MA, USA) equipped with an attenuated total reflection (ATR) accessory without a temperature controller. A small amount of the samples was evenly placed on the ZnSe ATR crystal to obtain the spectrum in the transmission mode ranging from 4000 cm^−1^ to 400 cm^−1^. The spectra obtained were processed using the latest OPUS software version 7.0.

### 3.7. Fluorescence Spectroscopy

Fluorescence spectroscopy has undergone rapid development due to its enormous technical advances, accuracy, and enhanced methods for analysis based on fluorophore compounds. The fluorescence spectra of protein extracted by using ultrasound and conventional alkaline extraction were taken using a FluoroMax4 Spectrofluorometer (HORIBA, Piscataway, NJ, USA) equipped with a xenon lamp. The method described by Mir et al. in 2019 was followed with little modification to acquire spectra. Spectra were taken at the fixed excitation wavelength of 220 nm for tyrosine, 260 nm for phenylalanine, and 280 nm for tryptophan, while the emission range was between 300 and 550 nm with a slit width of 1 nm. Three spectra were taken for each amino acid, and the average spectrum is presented in the results.

### 3.8. Experimental Design and Statistical Analysis

Based on the principle of central composite design (CCD), amplitude % (A), solute-to-solvent ratio (B), and pH (C) as the studied parameters for the maximum extraction of protein were optimized (Table 1) via response surface methodology (RSM) by using MATLAB (2007a). An analysis of variance (ANOVA) with a 95% confidence level was then carried out for each response variable in order to test the model significance and suitability.


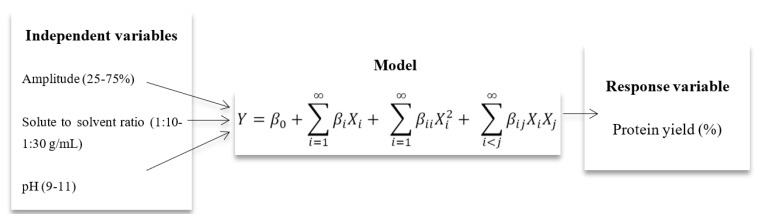
 where *Y* denotes the predicted value of the response variable; *β*_0_ denotes the intercepts; and *β_i_*, *β_ii_*, and *β_ij_* are the linear, second order, and interaction regression coefficients predictable by the model, respectively. *X_i_* and *X_j_* are the values of studied or independent variables.

## 4. Conclusions

This research revealed that ultrasonic-assisted extraction is significantly better and had beneficial effects in obtaining higher protein yields. During extraction, ultrasound power and pH were among the parameters which significantly affected the protein yield. This treatment altered the secondary and tertiary structure of MOSP, which were evident from FT-IR and fluorescence spectroscopy, respectively. Ultrasonication did not yield any degrading effects on MOSP in terms of functional properties. The functional properties of MOSP such as solubility, water- and oil-holding capacity, emulsion stability, and foaming capacity and stability improved in the beginning and then reduced as the amplitude of sonication further increased. These results would give an understanding of the mechanism and interrelation of structural changes with functional properties observed after the ultrasonic-assisted extraction of MOSP and its further application in the development of advanced sustainable food products.

## Figures and Tables

**Table 1:** Central composite design representing the experimental trials along with *M. oleifera* seed protein (MOSP) yield.

Run	Independent Variables (Coded Values)			Independent Variables (Actual Values)			Response (MOSP Yield (%))	
	A: Amplitude (%)	B: Solute-to-Solvent Ratio (g/mL)	C: pH	A: Amplitude (%)	B: Solute-to-Solvent Ratio (g/mL)	C: pH	Measured	Predicted
1	0	0	−1	50	1:20	9	34.76	34.77
2	1	−1	1	75	1:10	13	37.34	37.32
3	−1	0	0	25	1:20	11	33.15	33.13
4	−1	1	1	25	1:30	13	32.10	32.11
5 (c.p.)	0	0	0	50	1:20	11	36.62	36.59
6 (c.p.)	0	0	0	50	1:20	11	36.52	36.59
7	1	−1	−1	75	1:10	9	37.06	37.07
8	−1	−1	1	25	1:10	13	31.17	31.18
9	0	−1	0	50	1:10	11	36.22	36.20
10	1	0	0	75	1:20	11	39.30	39.29
11	1	1	1	75	1:30	13	38.28	38.29
12 (c.p.)	0	0	0	50	1:20	11	36.57	36.59
13	0	0	1	50	1:20	13	35.02	34.99
14	−1	1	−1	25	1:30	9	31.94	31.93
15	0	1	0	50	1:30	11	37.20	37.19
16	−1	−1	−1	25	1:10	9	30.92	30.91
17	1	1	−1	75	1:30	9	38.11	38.10

c.p. = central point.

**Table 2:** Analysis of variance (ANOVA) for quadratic model.

Source	Sum of Squares	df	Mean Square	F-Value	*p*-Value
Model	111.81	9	12.42	11,418.21	<0.0001 **
A: Amplitude	94.93	1	94.93	87,243.93	<0.0001 **
B: Solute-to-solvent ratio	2.42	1	2.42	2224.75	<0.0001 **
C: pH	0.1254	1	0.1254	115.29	<0.0001 **
AB	0.0002	1	0.0002	0.1838	0.6810 ^ns^
AC	0.0002	1	0.0002	0.1838	0.6810 ^ns^
BC	0.0050	1	0.0050	4.60	0.0693 ^ns^
A^2^	0.3720	1	0.3720	341.87	<0.0001 **
B^2^	0.0338	1	0.0338	31.11	0.0008 **
C^2^	7.81	1	7.81	7180.25	<0.0001 **
Residual	0.0076	7	0.0011		
Lack of Fit	0.0026	5	0.0005	0.2093	0.9308 ^ns^
Pure Error	0.0050	2	0.0025		
Cor Total	111.82	16			
*R* ^2^	0.9999				
*R*^2^ adjusted	0.9998				

** Significant at 0.01 level; ns = non-significant; df = degree of freedom.

**Table 3:** Coefficient estimation in terms of coded factors.

Factor	Coefficient Estimate	df	Standard Error	95% CI Low	95% CI High	VIF
Intercept	36.59	1	0.0141	36.55	36.62	
A: Amplitude	3.08	1	0.0104	3.06	3.11	1.0000
B: Solute to solvent ratio	0.4920	1	0.0104	0.4673	0.5167	1.0000
C: pH	0.1120	1	0.0104	0.0873	0.1367	1.0000
AB	0.0050	1	0.0117	−0.0226	0.0326	1.0000
AC	0.0050	1	0.0117	−0.0226	0.0326	1.0000
BC	−0.0250	1	0.0117	−0.0526	0.0026	1.0000
A^2^	−0.3726	1	0.0202	−0.4203	−0.3250	1.54
B^2^	0.1124	1	0.0202	0.0647	0.1600	1.54
C^2^	−1.71	1	0.0202	−1.76	−1.66	1.54

df = degree of freedom; CI = confidence interval; VIF = variance inflation factor.

**Table 4:** Functional properties of *M. oleifera* seed protein (MOSP) from conventional extraction (CE) and ultrasonic-assisted extraction (UAE).

Functional Properties	MOSP (CE)	MOSP (UAE)
Solubility (%)	5.56 ± 0.13	29.82 ± 0.21
WHC (g/g)	0.86 ± 0.009	1.02 ± 0.006
OHC (g/g)	0.91 ± 0.015	1.91 ± 0.013
Emulsion capacity (mg/mL)	58.39 ± 1.68	75.93 ± 1.19
Foaming capacity (%)	13.21 ± 0.27	24.23 ± 0.64

WHC = water-holding capacity; OHC = oil-holding capacity.

## Data Availability

Not applicable
